# Identification and validation of cellular senescence patterns to predict clinical outcomes and immunotherapeutic responses in lung adenocarcinoma

**DOI:** 10.1186/s12935-021-02358-0

**Published:** 2021-12-06

**Authors:** Weihao Lin, Xin Wang, Zhenyi Xu, Zhen Wang, Tiejun Liu, Zheng Cao, Xiaoli Feng, Yibo Gao, Jie He

**Affiliations:** 1 Department of Thoracic Surgery, National Cancer Center/National Clinical Research Center for Cancer/Cancer Hospital, Chinese Academy of Medical Sciences and Peking Union Medical College, Beijing, China; 2 Department of Epidemiology and Biostatistics, School of Public Health, Harbin Medical University, Harbin, China; 3 Department of Pathology, National Cancer Center/National Clinical Research Center for Cancer/Cancer Hospital, Chinese Academy of Medical Sciences and Peking Union Medical College, Beijing, China

**Keywords:** Cellular senescence, Lung adenocarcinoma, Prognosis, Tumor microenvironment, Immunotherapy

## Abstract

**Background:**

Aging and senescence can alter immune cell fitness and influence the efficacy of lung cancer treatments, especially immunotherapy. However, the correlations between cellular senescence and tumor microenvironment are still not clearly clarified and the value of cellular senescence-related genes in evaluating the immune infiltration and clinical outcomes of lung adenocarcinoma (LUAD) need further investigated.

**Methods:**

We identified three cellular senescence clusters by NMF algorithm and correlated the cellular senescence clusters with the immune landscape in LUAD patients. A prognostic scoring system was established using random survival forest algorithm and validated in 4 external cohorts. Multivariate Cox regression analysis was performed to evaluate the prognostic value of the scoring system. Expression of LYPD3 was evaluated by immunohistochemistry in LUAD samples.

**Results:**

Based on the mRNA expression profiles of 278 cellular senescence-related genes, three cellular senescence clusters with distinct prognosis were identified. We characterized three cellular senescence clusters by differences in biological processes, EMT score, expression of immunomodulatory genes, extent of intratumor heterogeneity and response to immunotherapy. Meanwhile, a cellular senescence-related scoring system (CSS) was established and validated as an independent prognostic factor and immunotherapy predictor of LUAD. Patients with low CSS was characterized by prolonged survival time. In response to anti-cancer drugs, patients with low CSS exhibited higher sensitivities to molecular drugs, such as Roscovitine (CDKs inhibitor), Lenaidornide (TNF-α inhibitor), MK2206 (Akt 1/2/3 inhibitor), and especially increased response to anti-PD-1/L1 immunotherapy.

**Conclusions:**

This study demonstrated the correlations between cellular senescence patterns and tumor immune landscape in LUAD, which enhanced our understanding of the tumor immune microenvironment and provided new insights for improving the outcome of immunotherapy for LUAD patients.

**Supplementary Information:**

The online version contains supplementary material available at 10.1186/s12935-021-02358-0.

## Introduction

The morbidity of lung cancer has already ranked the top 1 worldwide with an estimated 1.8 million deaths (18%) in 2020 [1]. According to the GLOBOCAN, 2020, cancer incidence increased with age and more than two thirds of patients with lung cancer are ≥ 65 years of age at the time of diagnosis [1, 2]. Accompanied with the trends of aging globally [3], aging lung cancer patients is growing over time. Moreover, the huge heterogeneity in aged patients was driven by a high variability of physiological age reflecting organ function deteriorations, immune function decline and cellular senescense [4], so that the treatment of elderly patients with non-small-cell lung cancer (NSCLC) represents still a challenge. Therefore, in the era of personalized cancer treatment [5], it is crucial to determine the cellular senescence factors associated with increasing age for the risk stratification of individualized treatments.

Emerging evidence suggests that aging is accompanied by cellular senescence, and manipulating cellular senescence biological processes can moderate or delay many aging-related diseases, such as cancer [6, 7]. Cellular senescence is a complex stress response that can be grouped into different categories including genome-based failures and signaling dysfunction [6, 8, 9], accompanied by numerous variations in gene expressions [10]. Recent studies clarified that aging and cellular senescence deeply modify the TME by facilitating the accumulation of many types of immunosuppressive cells [11], and the activation of a variety of danger-associated signaling molecules and cytokines [11–13], that will lead to extensive effects on the tumor microenvironment (TME) and tumor growth [14, 15]. In particular, aging and cellular senescence can alter immune cell fitness and, ultimately, have an impact on the efficacy of cancer treatments, especially immunotherapy [16, 17]. However, the correlations between cellular senescence and TME is still not clear clarified and the value of cellular senescence-related genes in evaluating the immune infiltrate of tumors and clinical outcomes need further investigated.

In our study, we comprehensively investigated the association between cellular senescence clusters and TME cell-infiltrating characteristics by analyzing the transcriptomic data of LUAD samples. We also constructed and validated a scoring system based on DEGs among the distinct clusters to predict overall survival (OS) and immune checkpoint blockade (ICB) therapy response for individual LUAD patients. Our findings pictured the linkage between cellular senescence patterns and TME in LUAD, and yielded insights into how cellular senescence might affect patient survival and immunotherapy, which may provide assistance to individualized therapeutic strategies.

## Methods

### Public cohorts and clinical specimens

A total of 1069 patients with both available transcriptomics data and corresponding clinical information were included in our study. Data of 500 patients obtained from The Cancer Genome Atlas (TCGA, https://portal.gdc.cancer.gov) was used as the training cohort. Fragments per kilobase million (FPKM) value of the TCGA cohort were then transformed into transcripts per million (TPM) value before further analysis. Datasets GSE30219 [18] (n = 83), GSE31210 [19] (n = 226), GSE37745 [20] (n = 106) and GSE50081 [21] (n = 127) were downloaded the Gene Expression Omnibus (GEO, https://www.ncbi.nlm.nih.gov/geo) database via the R package ‘GEOquery’ and the mean expression values were used when genes matched with multiple probes for these microarray data. We also collected one dataset of NSCLC patients treated with anti-PD-1/PD-L1 immunotherapy (GSE135222 [22], n = 27) from GEO database. The clinicopathological data of the enrolled patients in public cohorts were shown in Additional file 8: Table S1.

Moreover, we also retrospectively collected 74 surgically resected, formalin-fixed, paraffin-embedded lung adenocarcinoma tissues and 74 adjacent normal tissues from the biobank of National Cancer Center/National Clinical Research Center for Cancer/Cancer Hospital in Chinese Academy of Medical Sciences and Peking Union Medical College (Beijing, China) and constructed a tissue microarray (TMA). Patients enrolled were informed consent and this study was approved by the Ethics Committee of the National Cancer Center/Cancer Hospital, Chinese Academy of Medical Sciences, and Peking Union Medical College. The clinicopathological data of the enrolled patients in our cohort were shown in Additional file 9: Table S2.

### Identification of cellular senescence clusters by NMF

A non-negative matrix factorization (NMF) [23] clustering algorithm was implemented to identify distinct cellular senescence clusters based on the expression profiles of 74 prognostic cellular senescence-related genes. The “brunet” option was chosen and 100 iterations were performed for NMF. The optimal clustering number was determined according to the cophenetic, dispersion, and silhouette coefficients. The NMF clustering was carried out using the R package ‘NMF’.

### Construction of cellular senescence score (CSS)

To construct a cellular senescence-related scoring system, we first determined the differentially expressed genes (DEGs) among distinct cellular senescence clusters using the R package ‘limma’, with the threshold set at adjusted *P* < 0.001. A total of 366 DEGs were screened out. Subsequently, DEGs related to prognosis were identified through univariate Cox regression analysis, with the threshold set at *P* < 0.01. Random survival forest analysis with the R package ‘randomForestSRC [24]’ was performed to further narrow the candidate genes. The top 10 genes sorted by importance were chosen to perform combinations analysis. For each combination among the 1023 combinations generated by the 10 genes, a multivariate Cox regression model was constructed and risk scores were calculated based on the expression values of each gene and its corresponding multivariate Cox regression coefficient. Patients were stratified into high- and low-risk group according to the median value of risk score and next Kaplan Meier analysis was conducted. Finally, the combination with the smallest *P* value were considered as the cellular senescence-related scoring system.

### Enrichment analysis

Gene Ontology (GO) [25] annotation for cellular senescence-related DEGs was performed using the R package ‘clusterProfiler [26]’. Gene Set Enrichment Analysis (GSEA) was conducted to identified the differences in biological processes between CSS-high and CSS-low groups using the javaGSEA desktop application (GSEA 4.1.0 [27]). Moreover, Gene set variation analysis (GSVA) was also carried out with the R package ‘GSVA [28]’. The gene sets of “h.all.v7.2.symbols” downloaded in MSigDB and the known gene sets constructed by Mariathasan et al. [29] were used for GSVA enrichment analysis.

### Evaluation of immune infiltration, key immune characteristics and EMT score

Single sample gene set enrichment analysis (ssGSEA) was implemented to estimate immune cell abundance of each sample in TCGA cohort based on a gene panel [30] marking 28 immune cell types. Besides, key immune characteristics [31], including leukocyte fraction, stromal fraction, SNV neoantigen, Indel neoantigen, intratumor heterogeneity (ITH), TCR Shannon and BCR Shannon were downloaded from the following website: https://gdc.cancer.gov/about-data/publications/panimmune and were compared among distinct cellular senescence clusters or between CSS-high or CSS-low group. The EMT score was calculated based on the epithelial-to-mesenchymal transition gene signature, which included 25 epithelial and 52 mesenchymal marker genes. The formula of the EMT score was described in previous studies [32, 33].

### Prediction of immunotherapeutic response and drug sensitivity

The Tumor Immune Dysfunction and Exclusion (TIDE, http://tide.dfci.harvard.edu/) [34, 35] and immunophenoscore (IPS, https://tcia.at) [30], which were considered as superior predictor of immunotherapy response, were used to predict responses to immunotherapy among distinct cellular senescence clusters and between CSS-low and CSS-high groups. Patients’ half-maximal inhibitory concentration (IC50) to 138 drugs were also quantified based on the Genomics of Drug Sensitivity in Cancer (GDSC, https://www.cancerrxgene.org) [36] database using the R package ‘pRRophetic [37]’.

### Immunohistochemistry (IHC) staining and evaluation

The tissue microarray slide was immunostained with a rabbit antibody against human LYPD3 (abcam; ab151709). The immunohistochemical staining was evaluated based on the percentage of positive cells and the staining intensity. The expression ratio was scored as 1 (0–25%), 2 (26–50%), 3 (51–75%) or 4 (76–100%), while signal intensity was scored as 0 (negative), 1 (weak), 2 (moderate) or 3 (strong). The final score of each sample was obtained by multiplying the expression ratio and the signal intensity. All the immunostained tissues were blindly reviewed by two pathologists.

### Statistical analysis

R software (version 3.6.1), GraphPad Prism 8.0 and Stats 16.0 were used to analysis data and plot graphs. Wilcoxon test and Kruskal–Wallis test were applied for comparisons of two and three groups, respectively. The overall survival probability of patients was analyzed by Kaplan–Meier method and log-rank test. Multivariate survival analysis was carried out using the Cox regression model. Meta-analysis was performed to assess the prognostic value in the pooled cohort. The R package ‘rms’ and ‘nomogramEx’ were applied to construct the nomogram adopting variables including age, gender, T stage, N stage, TNM stage as well as CSS. Calibration curves were utilized to assess the consistency between actual and predicted survival time. Time-dependent receiver operating characteristic (ROC) curves were generated to compare the predictive accuracy of different survival factors. Significantly mutated genes between CSS-high and CSS-low groups and the interaction effect of mutated genes were analyzed by R package ‘maftools [38]’. Two-sided *P* < 0.05 was considered statistically significant.

## Result

### Identification of distinct cellular senescence molecular patterns in LUAD

**Fig. 1:**
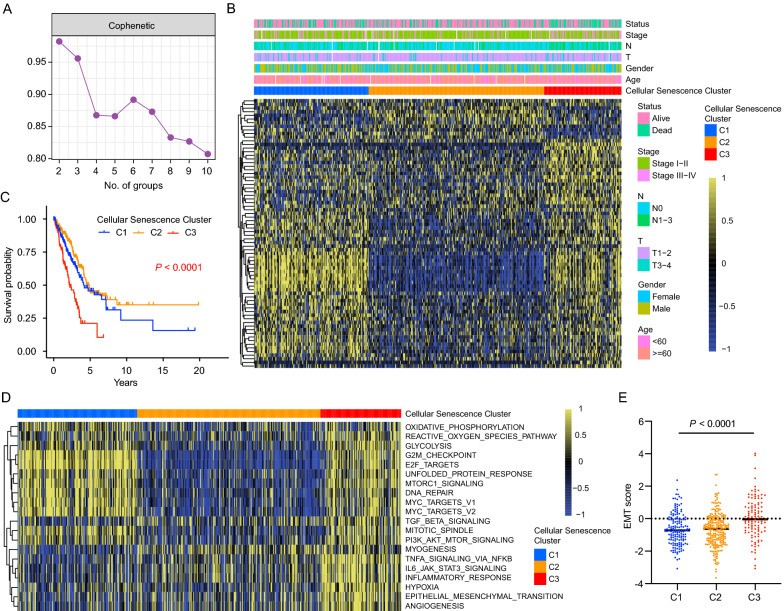
Cellular senescence-related molecular patterns with distinct prognosis and biological characteristics in LUAD. **A** Cophenetic correlation from NMF analysis of LUAD tumors. **B** Heatmap of the 74 cellular senescence-related prognostic genes in TCGA cohort. The age, gender, TNM stage, and survival status of TCGA LUAD cohorts were used as patient annotations. **C** Kaplan–Meier survival curves of LUAD patients in the three distinct clusters (P < 0.0001, Log-rank test). **D** GSVA enrichment analysis of Hallmark pathways in distinct cellular senescence clusters. Yellow indicated activated pathways, and blue indicated inhibited pathways. **E** The distribution of EMT scores in different cellular senescence clusters. The statistical difference of three gene clusters were compared through the Kruskal–Wallis test. (P < 0.001)

To characterize cellular senescence molecular patterns and screen out potential targets, we developed a workflow as shown in Additional file 1: Fig. S1. A manually-curated gene list including 278 cellular senescence-related genes was extracted from the CellAge database (Additional file 10: Table S3). To comprehensively explore the expression patterns of the cellular senescence-related genes (SRGs) in LUAD, a total of 1069 patients from 6 cohorts (TCGA cohort, GSE30219, GSE31210, GSE37745, GSE50081 and GSE135222) with corresponding clinical information were enrolled. Univariate Cox regression analysis showed that 74 of 278 cellular senescence-related genes were correlated with LUAD prognosis (*P* < 0.05, Additional file 10: Table S4) in TCGA cohort. Based on the expression profiles of the prognostic genes, we stratified patients into three distinct clusters through a nonnegative matrix factorization (NMF) algorithm (156 cases in Cluster 1 (C1), 239 cases in Cluster 2 (C2) and 105 cases in Cluster 3 (C3), Fig. 1A and Additional file 2: Fig. S2). The clinical information and gene expression of three cellular senescence clusters were displayed in Fig. 1B, and we found that patients with advanced clinical stage and lymph node metastasis (N1–3) were mainly concentrated in C3 subgroup (Fig. 1B), suggesting the prognostic differences among distinct cellular senescence clusters in LUAD. As expected, patients in C3 did exsert a significant worst survival (Fig. 1C, Log-rank test, *P* < 0.0001) compared with C1 and C2.

To further investigate the biological processes underlying three distinct cellular senescence clusters, GSVA enrichment analysis was performed. As shown in Fig. 1D, Cluster 1 and Cluster 3 was significantly enriched in carcinogenic activation and tumor proliferation related pathways, including E2F_target, G2M_checkpoint and MYC_target. Intriguingly, Cluster 3 also presented enrichment pathways prominently associated with stromal activation, such as TGF-β signaling, angiogenesis, hypoxia and EMT pathways, suggesting an immunosuppressive tumor microenvironment in samples of Cluster 3. Additionally, based on the gene expression of epithelial markers and mesenchymal markers, we calculated the EMT score of each sample and found a significantly higher EMT score in Cluster 3 (Fig. 1E), further confirmed the activation of EMT in samples of Cluster 3.

### Characterization of immune landscape in distinct cellular senescence clusters

**Fig. 2:**
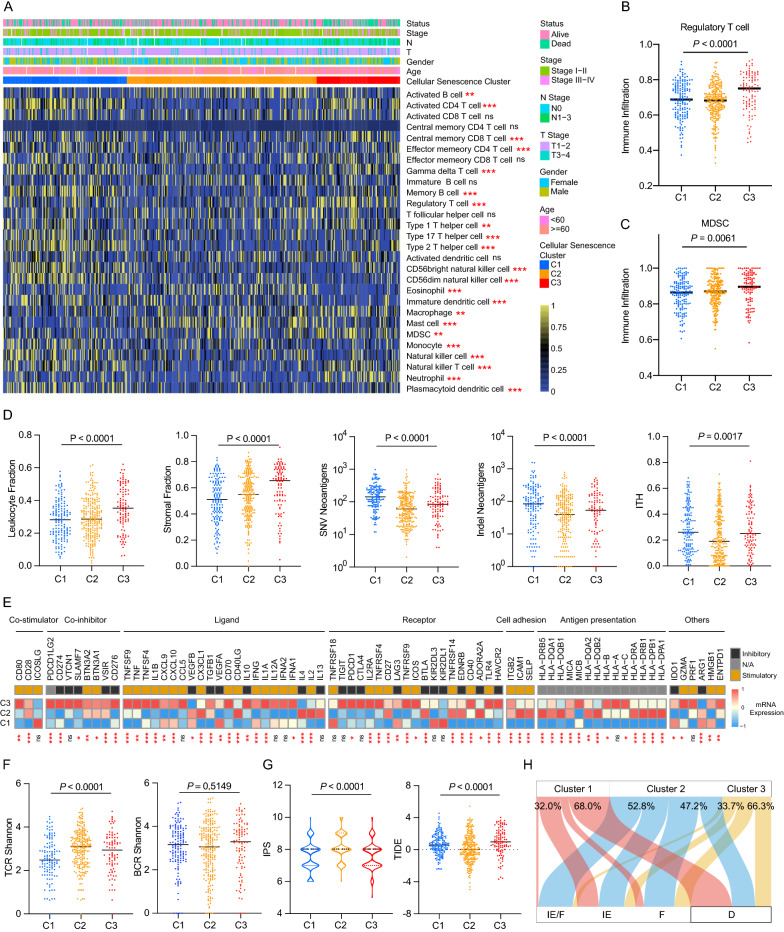
The immune landscape in distinct cellular senescence-related molecular patterns in LUAD. **A** Single-sample gene set enrichment (ssGSEA) analysis identifying the relative infiltration level of immune cell populations in three cellular senescence clusters of LUAD samples in TCGA cohort. (B-C) Differences in regulatory T cell (**B**) and MDSC (**C**) proportion among distinct cellular senescence patterns in TCGA cohorts. **D** The relative distributions of leukocyte fraction, stromal fraction, SNV neoantigens, Indel neoantigens, and ITH score were compared among three cellular senescence clusters. **E** Heatmap depicting the mean values of mRNA expressions of immune-related genes among distinct clusters. **F**–**G** Comparisons of TCR Shannon, BCR Shannon (**F**), IPS and TIDE score (**G**) among three cellular senescence clusters. The statistical difference of three clusters were compared through the Kruskal–Wallis test. **G** Alluvial diagram showing the relations of cellular senescence clusters (upper) and TME subtype (lower) across LUAD patients. The statistical difference of three clusters were compared through the Kruskal–Wallis test. *ns* not significant; *P < 0.05; **P < 0.01; ***P < 0.001

Previous studies have revealed the association between cellular senescence and immune infiltration in multiple cancer types [14, 15, 39]. Thus, we applied ssGSEA algorithm to determine the relative abundance of 28 immune infiltrating cells in each sample and compared the component difference of immune cells among distinct cellular senescence clusters (Fig. 2A). Particularly, we found that regulatory T cells (Tregs) and Myeloid-derived suppressor cells (MDSCs) were markedly elevated in Cluster 3 (Fig. 2B, C), which was concordant with previous observations linking Cluster 3 to an immunosuppressive phenotype. In addition, we explored the immune characteristics in distinct cellular senescence clusters. As shown in Fig. 2D, both leukocyte and stromal fractions were increased in Cluster 3. Cluster 1 had the highest SNV neoantigens, Indel neoantigens and ITH, while Cluster 2 was defined by the lowest neoantigens and ITH.

Immunomodulators play a critical role in shaping the tumor microenvironment (TME) and cancer immunotherapy. Therefore, to further investigate the complex crosstalk of immunomodulators, immune infiltration and cellular senescence, we explored the expression of immunomodulators in different clusters. As depicted in Fig. 2E, many inhibitory immune checkpoint genes, including PD-L1, PD-1 and TIM3 were markedly upregulated in C3, whereas most major histocompatibility complex (MHC) class II genes were highly expressed in C2. Moreover, we observed significantly loss of both MHC I and MHC II machinery in C1 samples, suggesting that the disruption of productive tumor neoantigens presentation could facilitate immune evasion in LUAD patients of Cluster 1 [40]. Next, we analyzed the TCR and BCR Shannon among different clusters. Our results show that TCR Shannon was significantly higher in C2, while no difference in BCR Shannon was observed among different clusters (Fig. 2F). Finally, we used TIDE and IPS score to evaluate the clinical efficacy of immunotherapy among three clusters. In our results, Cluster 2 had the lowest TIDE score, and the highest IPS, implying that patients in Cluster 2 could benefit more from immunotherapy than Cluster 1 and 3 (Fig. 2G).

Recently, a large-scale transcriptomic analysis [41] identified four distinct tumor microenvironment (TME) subtypes, including immune-enriched, fibrotic (IE/F), immune-enriched, non-fibrotic (IE), fibrotic (F), and immune-depleted (D), which were conserved across 20 different cancers. In lung cancer, immunotherapy-treated patients with immune-enriched TME subtypes IE/F and IE demonstrated longer OS than those with subtypes F and D. We focused on the distribution of TME subtypes in cellular senescence clusters. In our study, both Cluster 1 and Cluster 3 comprised over 65% of F and D samples, whereas Cluster 2 comprised over 50% of IE/F and IE samples (Fig. 2H), which were in line with our hypothesis that patients in Cluster 2 might response better to immunotherapy compared to those in Cluster 1 and Cluster 3.

Taken together, our comprehensive analysis revealed that cellular senescence clusters were significantly correlated with patients’ prognosis and TME characteristics, which might provide new insights on LUAD classification system.

### Construction of the cellular senescence score for overall survival in LUAD patients

**Fig. 3:**
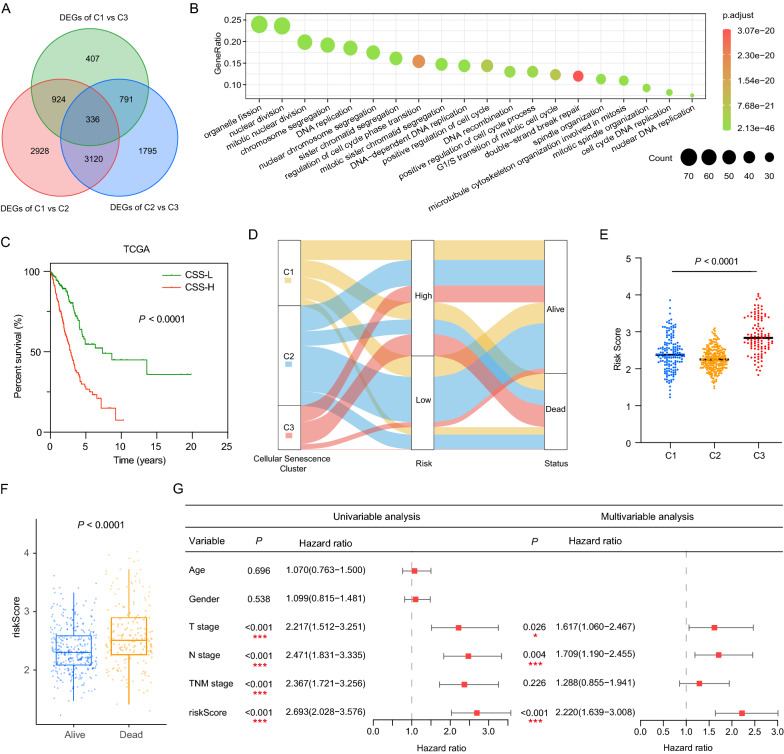
Construction of cellular senescence score (CSS) and impact of CSS on clinical outcome for LUAD patients. **A** 336 cellular senescence clusters-related differentially expressed genes (DEGs) among three cellular senescence clusters were shown in the Venn diagram. **B** GO enrichment analysis of the 336 DEGs. **C** Kaplan–Meier curves for patients with high and low cellular senescence score. **D** Alluvial diagram showing the relations of cellular senescence clusters, cellular senescence score (CSS) and survival status of LUAD patients in TCGA cohort. **E** Comparison of cellular senescence score among three cellular senescence clusters. **F** The relative distributions of CCS between alive and dead patients in TCGA cohort. **G** Forrest plot of the univariate and multivariate Cox regression model analysis, which included factors of patients’ age, gender, TNM stage, and CSS in the TCGA cohort. *ns* not significant; *P < 0.05; **P < 0.01; ***P < 0.001

To further reveal the underlying biological role of cellular senescence clusters, we identified 336 overlapping cellular senescence molecular pattern-related DEGs under a threshold of *adj. P* < 0.001 (Fig. 3A; Additional file 10: Table S5). GO enrichment analysis for DEGs revealed that enrichment of biological processes notably related to cell cycle, DNA replication and DNA damage repair, which confirmed the important role of cellular senescence in tumor progression (Fig. 3B).

Given the heterogeneity and complexity of cellular senescence in LUAD individuals [10], we sought to establish a cellular senescence-related scoring system for clinical prognosis prediction. We first evaluated the prognostic value of the 336 DEGs using univariate Cox regression analysis. With a threshold of *P* < 0.01, a total 194 genes (Additional file 3: Fig. S3A, Additional file 10: Table S6) were screened out as promising candidates. Random survival forest analysis was then implemented to further shrink the scope of candidate genes. The top 10 genes sorted by importance (Additional file 3: Fig. S3B) were subsequently submitted to the Kaplan–Meier analysis of combination. Among the 1023 combinations, a combination of 4 genes (C1QTNF6, SQOR, LYPD3 and FAM83B) obtained the minimum *P* value (Additional file 3: Fig. S3C), which was integrated to build cellular senescence scoring system. Finally, based on the multivariate Cox regression, the cellular senescence score (CSS) was constructed and the risk score of each patient were calculated as follows: CSS = (0.2921 × expression value of C1QTNF6) + (0.1810 × expression value of SQOR) + (0.0524 × expression value of LYPD3) + (0.1285 × expression value of FAM83B). LUAD samples were stratified into CSS-high and CSS-low groups according to the optimal cutoff value of the risk score. Kaplan–Meier analysis demonstrated that patients with high CSS exhibited worse prognosis than those with low CSS (HR = 2.775, 95% CI 2.073–3.714, log-rank *P* < 0.0001, Fig. 3E). The area under the ROC curves for 2-, 3-, 5-year survival time were 0.690, 0.696 and 0.638, respectively, implying that CSS was a reliable predictive model (Additional file 3: Fig. S3D).

Relationship of attributes including cellular senescence clusters, risk groups and survival status of patients was illustrated in an alluvial diagram (Fig. 3D). Consistent with the above findings, 87.5% of patients in cluster 3, which was relevant to the worse survival outcome, were classified into high-risk group, while the CSS-low subgroup comprised 63.8% of C2 patients and only 5.8% of C3 patients (Fig. 3D). Therefore, it was not surprising that cluster 3 had the highest risk score (Fig. 3E). In addition, the risk score was also significantly increased in the dead patients compared with living patients (Fig. 3F).

### CSS serves as an independent prognostic factor for LUAD

**Fig. 4:**
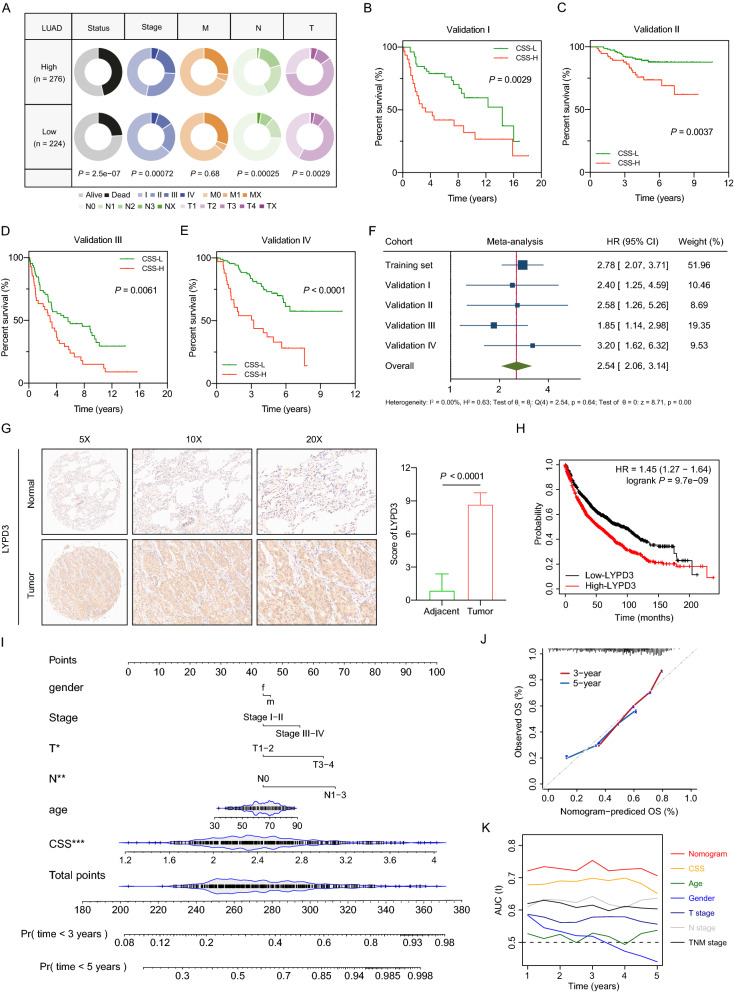
Evaluation of CSS performance in independent datasets and construction of a CSS-based nomogram. **A** Comparisons of clinical features between CSS-low and CSS-high subgroups. **B**–**E** Kaplan–Meier curves for patients with high and low CCS in validation I(B), II(C), III(D), IV(E) cohorts. **F** Meta-analysis. **G** Representative images (left) and score comparison (right) of IHC staining for LYPD3 in adjacent non-tumor tissues and LUAD tissues. **H** Survival analysis comparing low and high expression levels of LYPD3 in the Kaplan–Meier plotter database. **I** Construction of a nomogram combining the CCS and clinical features for prediction of OS. **J** Calibration analysis indicated a high accuracy of OS prediction. **K** AUC plotted for different durations of OS for nomogram-based signature, CSS, Age, T stage, N stage and TNM stage in LUAD patients in TCGA cohort

To investigate the independent prognostic value of CSS, we performed multivariate Cox regression analysis considering clinical features including patients’ age, gender, T stage, N stage, TNM stage), which demonstrated that CSS (HR = 2.220, 95% CI 1.639–3.008, *P* < 0.001) could serve as an independent prognostic risk factor for overall survival of LUAD patients in TCGA cohort. (Fig. 3G). To further explore the clinical relevance of CSS in depth, we compared the distribution of several clinicopathological features between CSS-high and CSS-low groups. Significantly distribution differences of survival status (*P* = 2.5e−07), TNM stage (*P* = 0.0007), T stage (*P* = 0.0029) and N stage (*P* = 0.0003) were observed between distinct subgroups in TCGA cohort (Fig. 4A). Patients with different stages of lung adenocarcinoma have different prognosis and treatment strategies [42], thus, we applied CSS in patients with different stage in the training cohort. As depicted in Additional file 4: Fig. S4A, high CSS was associated with poor prognosis despite of patients’ stage. CSS also discriminated high risk patients with worse prognosis in different subgroups with different clinicopathological features, including T stage (Additional file 4: Fig. S4B), N stage (Additional file 4: Fig. S4C), age (Additional file 4: Fig. S4D), and gender (Additional file 4: Fig. S4E).

To confirm the prognostic robustness of the cellular senescence score, CSS was further validated in four independent cohorts described previously. Consistent and significant differences were observed in all validation cohorts (Fig. 4B–E), including Validation cohort I (HR = 2.398, 95% CI 1.252–4.592, log-rank *P* = 0.0029), Validation cohort II (HR = 2.578, 95% CI 1.264–5.258, log-rank *P* = 0.0037), Validation cohort III (HR = 1.846, 95% CI 1.145–2.977, log-rank *P* = 0.0061) and Validation cohort IV (HR = 3.198, 95% CI 1.619–6.317, log-rank* P* < 0.0001). Meta-analysis was also performed to comprehensively assess the prognostic value of CSS in the pooled cohort which integrated the training cohort and four validation cohorts (Fig. 4F). Our result revealed that patients in the CSS-high group showed unfavorable OS compared to those in CSS-low group (pooled HR = 2.54, 95% CI 2.06–3.14, *P* < 0.0001). Collectively, CSS exhibited as a reliable and accurate prognostic factor for evaluating LUAD patient outcomes.

Consistently, overexpression of C1QTNF6, SQOR, LYPD3 and FAM83B were associated with worsened prognosis in patients with LUAD in terms of OS (Additional file 5: Fig. S5A–D). However, only LYPD3 was dramatically overexpressed in tumor tissues compared with normal tissues (Additional file 5: Fig. S5E, *P* < 0.05) in GEPIA2 [43], which combined TCGA and GTEx data together to assess the gene expression between normal and tumor tissues more accurately. Therefore, we gave our attention to LYPD3. We analyzed the protein expression of LYPD3 on a tissue microarray using immunohistochemical (IHC) staining. As shown in Fig. 4G, the protein expression level of LYPD3 was remarkedly elevated in tumor samples compared to that in adjacent normal tissues. We also performed survival analysis on Kaplan–Meier plotter database [44], which integrated multiple cohorts. The result showed that LUAD patients with high LYPD3 level had a significantly worse prognosis than those with low LYPD3 (Fig. 4H), suggesting that LYPD3 might serve as a potential prognostic marker in LUAD.

### Construction of integrated models to optimize risk stratification and survival prediction in LUAD patients

To better predict the survival probability for LUAD patients, a predictive nomogram was built based on the integration of CSS and other clinicopathological features, including age, gender, T stage, N stage and TNM stage (Fig. 4I). The calibration curves of the nomogram for 3-years and 5-years survival probability showed outstanding consistency with the ideal performance, suggesting a high accuracy of our nomogram (Fig. 4J). Additionally, compared with other clinicopathological features (age, gender, T stage, N stage and TNM stage), the CSS-based nomogram exhibited the best ability at predicting the OS, with an average AUC above 0.7 (Fig. 4K).

### Comprehensive analyses of tumor mutations and enriched biological pathways between different risk groups

**Fig. 5:**
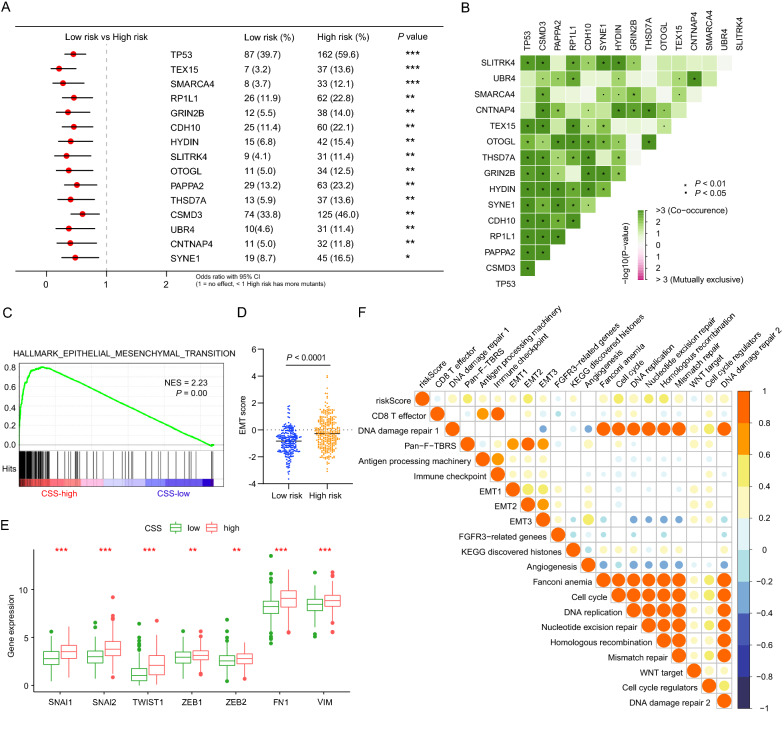
Characteristics of tumor mutations and biological pathways between CSS-high and CSS-low subgroups. **A** Comparison of differential mutated genes between CSS-low and CSS-high subgroups. **B** Interaction effect of differential mutated genes in patients in CSS-low and CSS-high subgroups. **C** GSEA analysis showed that the EMT pathway was activated in CSS-high subgroup in LUAD. **D**–**E** EMT score (**D**) and differences in EMT hub genes including SNAI1, SNAI2, TWIST1, ZEB1, ZEB2, FN1 and VIM (**E**) between CSS-high and CSS-low subgroups. **F** Correlations between CCS and the known gene signatures in LUAD patients. Positive and negative correlation were marked with orange and blue, respectively; *ns* not significant; *P < 0.05; **P < 0.01; ***P < 0.001

To investigate the potential mechanisms leading the distinct outcomes between CSS-high and CSS-low groups, we first compared the mutant frequency between the two groups. As illustrated in Fig. 5A, the top 15 differentially mutated genes, including TP53, TEX15, SMARCA4, RP1L1, GRIN2B, CDH10, HYDIN, SLITRK4, OTOGL, PAPPA2, THSD7A, CSMD3, UBR4, CNTNAP4 and SYNE1, were all mutated more frequently in the CSS-high group. Moreover, significant co-occurrences were also observed among mutations of the 15 genes (Fig. 5B). Next, we performed GSEA with annotation of HALLMARK gene sets and found significant enrichment of epithelial mesenchymal transition (EMT) pathway in CSS-high group (Fig. 5C). Consistently, higher EMT score and gene expression of EMT markers including SNAI1, SNAI2, TWIST1, ZEB1, ZEB2, FN1, and VIM were also observed in high-risk group (Fig. 5D, E). To further confirm the results, we also carried out correlation analysis between CSS and the known signatures. Intriguingly, CSS were negatively correlated with CD8 T effector pathway, while positively correlated with EMT and pan-fibroblast TGF-β response signature (Pan-F-TBRS) pathway, which served as the measurement of TGF-β pathway activity specifically in fibroblasts (Fig. 5F). EMT and TGF-β pathway, which were considered as T cell suppression, was shown to strongly connected with CSS, prompting us to further investigate the connection between CSS and the tumor immune microenvironment.

### CSS was associated with tumor immune infiltration and predicted therapeutic benefits

**Fig. 6:**
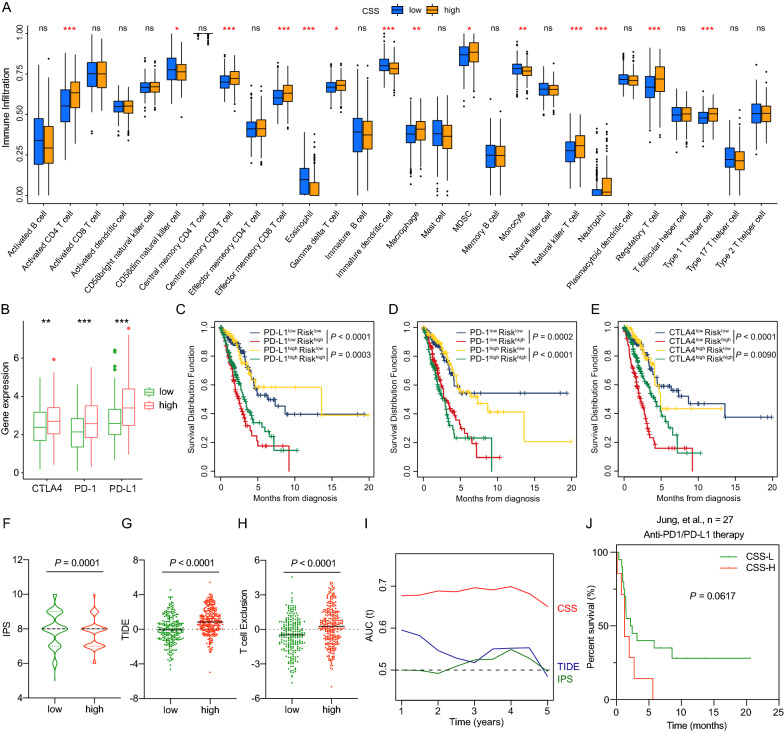
Response and survival predictive performance of CSS in immunotherapy. **A** The fraction of tumor-infiltrating immune cells between CSS-low and CSS-high groups in TCGA cohort by ssGSEA. **B** Comparison of checkpoints genes expressions (PD-L1, PD-1, and CTLA4) between CSS-low and CSS-high groups. **C**–**E** Kaplan–Meier curves for four patient groups stratified by CCS and immune checkpoint genes (PD-L1 (**C**), PD-1 (**D**), CTLA4 (**E**)). **F**–**H** IPS(F), TIDE score (**G**) and T cell Exclusion score (**H**) were compared between CSS-low and CSS-high subgroups in TCGA cohort. **I** The comparison of AUC of CSS, TIDE score, and IPS for OS in TCGA cohort. **J** Kaplan–Meier survival curves for patients with low and high CSS in anti-PD-L1 or anti-PD1-treated lung cohort. The statistical differences between two groups were compared through the Wilcoxon test. *ns* not significant; *P < 0.05; **P < 0.01; ***P < 0.001

To drive deeper in relationship between CSS and tumor immunity, we first compared the abundance of infiltrated immune cells between high- and low risk groups. Interestingly, our data showed that CSS-high group had significantly higher infiltration level of immunosuppressive cells, including MDSCs and Tregs (Fig. 6A), which was in accordance with our hypothesis that patients in high-risk group might be under an immunosuppressive state. Subsequently, we explored the expressions of immune checkpoint genes in different groups. Increasing expression of PD-L1, PD-1, CTLA4 was observed in high-risk group (Fig. 6B), meanwhile, co-inhibitory immune checkpoint molecules, including TIM3, LAG3, etc. which drive T-cell exhaustion, were also significantly upregulated in CSS-high group (Additional file 6: Fig. S6A). Taken together, these results indicated that patients in high-risk group exhibited inertia in antitumor immunity, which might contribute to their unfavorable outcomes.

We hypothesized that combination of CSS with immune checkpoint genes or TMB would further improve the ability to predict prognosis of LUAD. Thus, to explore the effect of CSS in concert with immune checkpoint genes (PD-L1, PD-1, CTLA4) on LUAD patients’ outcomes, survival analysis of four patient groups stratified by CSS and expression of immune checkpoint genes were conducted. As expected, patients with low CSS and low PD-L1 exhibited significantly prolonged survival than those with high CSS and low PD-L1, and patients with low CSS and high PD-L1 were also linked with favorable OS compared to those with high CSS and high PD-L1 (Fig. 6C). Similarly, patients in CSS-low group tended to have significantly favorable survival prospects relative to those in CSS-high group regardless of whether the expression of PD-1 or CTLA4 was high or low (Fig. 6D, E). Given that the critical role of TMB in clinical practice, we also compared the distribution of TMB between subgroups. Patients with high CSS showed a higher TMB than those with low CSS (Additional file 6: Fig. S6B). Noticeable survival differences were found between high- and low- risk groups in patients with low TMB (Additional file 6: Fig. S6C). These observed connections between CSS and immune checkpoint genes/TMB indicated that the combination of CSS with immune checkpoints or TMB showed better prognosis stratifications and CSS might serve as a potential predictor of treatment response in immunotherapy.

Newly identified predictors, including IPS and TIDE, were widely applied and considered as superior biomarkers to evaluate immunotherapy response. Thus, to elucidate the effects of CSS in immunotherapy, we extended our analysis to compare the distribution of the known markers in low- and high- risk groups. Our results revealed that IPS was significantly elevated in CSS-low group, while TIDE and T cell exclusion score were decreased in CSS-low group (Fig. 6F–H). We also compared the time-dependent AUC value of CSS with those of TIDE and IPS, which showed that CSS performed better than TIDE and IPS in predicting OS of LUAD patients (Fig.  6I). The practicability of CSS for predicting the therapeutic benefit was further analyzed using independent cohort of anti-PD-L1 or -PD1-treated patients with lung cancer [22]. Patients with low CSS distinctly exhibited longer survival time compared to those with high CSS (*P* = 0.0617, Fig. 6J). These results implied that CSS could function as an indicator of immunotherapy and low CSS may correlate with a favorable response to immune checkpoint inhibitors (ICIs) therapy.

To further discover the potential therapy targets, we screened the IC50 values of 138 compounds from GDSC in LUAD patients, and a total of 16 drugs were screened out (*P* < 0.05). We found that patients in low-risk group were more sensitive to all the 16 drugs including CCT007093 (protein phosphatase 1D inhibitor), VX.702 (p38 MAP kinase inhibitor), Roscovitine (CDKs inhibitor), Lenaidornide (TNF-α inhibitor), MK2206 (Akt 1/2/3 inhibitor) etc., providing new perspective for drug development (Additional file 7: Fig. S7).

## Discussion

To date, since the immune checkpoint inhibitors (ICIs) targeting programmed cell death-1/ligand-1 (PD-1/PD-L1) and cytotoxic T lymphocyte antigen-4 (CTLA-4) have led to a paradigm shift in lung cancers [45, 46], the efficacy of ICIs on older patients remains controversial as old persons inclusion is lacking in current clinical trials [47]. Additionally, the immunotherapy of LUAD has proven a challenge for the era of personalized therapy due to inter- and intra-tumoral heterogeneity. Therefore, studies on the biological mechanisms and prognostic biomarkers of LUAD concerning cellular senescence-related genes may offer an opportunity to identify lung cancer subtypes, improving the future application of precision-focused treatments for LUAD. In the present study, we comprehensively analyzed the role of distinct cellular senescence clusters in clinical implication and immune landscape in LUAD. Moreover, we constructed an independent prognostic model based on cellular senescence cluster-related DEGs, which might contribute to make personalized immunotherapy strategies for oncologists.

Transcriptomic analyses pave a way to discriminate the heterogeneity and complexity of tumors and to discover novel prognostic and predictive biomarkers for novel therapeutic strategies [48]. Intriguingly, we first identified three different cellular senescence clusters with distinct clinical outcomes and TME characteristics in LUAD patients. The findings indicated that cellular senescence-related genes may play crucial roles in the generation of different biological process and immune phenotypes, which were correlated with diverse tumorigenesis and anticancer immunity in individual tumors. For example, cellular senescence cluster 3 was significantly enriched in carcinogenic activation pathways, meanwhile also associated with stromal activation pathways, including TGF-β signaling, angiogenesis, hypoxia and EMT pathways, indicating that patients in cluster 3 might have an immunosuppressive tumor microenvironment, which facilitated tumor cell invasion into the stroma, angiogenesis and tumor development [29, 49, 50]. Intriguingly, patients in cluster 3 had the highest proportion of Tregs and MDSCs characterized by the suppressive immunity [51, 52]. On the contrary, cluster 2 had the lowest ITH and TIDE score, as well as the highest IPS and TCR Shannon, indicating the most beneficial from immunotherapy for patients in cluster 2 [30, 34, 35, 53–55]. Moreover, the identified cellular senescence clusters presented consistency with the published TME subtypes that patients with immune-enriched TME subtypes IE/F and IE demonstrated better survival than those with subtypes F and D [41]. Taken together, the newly identified cellular senescence clusters might provide novel insights on classification system of LUAD.

Through random survival forest algorithm, we constructed a novel survival prediction model (CSS) based on the expression of four cellular senescence cluster-related DEGs (C1QTNF6, SQOR, FAM83B and LYPD3), which performed well in four external validation cohorts across different platforms. Furthermore, a nomogram combined CSS and clinicopathological factors was generated to quantify risk evaluation and survival probability. Compared to other traditional features, the CSS-based nomogram presented the best accuracy and discrimination in survival prediction. Notably, our results implied that the CSS was an independent prognostic factor for LUAD patients, additionally functioned as a predictor of immunotherapy. It is suggested that low CSS may correlate with a favorable response to ICI therapy and CSS coupled with specific immune checkpoint factors or TMB might serve as predictive biomarkers of ICIs response and prognosis. To investigate the possible mechanisms underlying the predictive role of CSS, we uncovered that CSS-high group had significantly higher infiltration level of immunosuppressive cells, including MDSCs and Tregs. Furthermore, CSS were also negatively correlated with CD8 + T effector pathway, meanwhile positively correlated with EMT and Pan-F-TBRS pathway, delineating the immunosuppressive microenvironment and the dysfunctional immune reaction [31]. Thus, further studies will be important to decipher whether cellular senescence molecular determinants reshape tumor microenvironments and affect the prognosis and immunotherapy response of LUAD patients.

Among the four signature genes, only expression of LYPD3 was abnormally elevated in tumor samples compared with normal tissues in GEPIA2, which was further confirmed by IHC in our TMA. Consistently, previous studies also showed that LYPD3 (C4.4A) was overexpressed in lung cancer and played as a tumorigenesis and metastasis-associated cell surface protein [56, 57]. Recently a phrase I clinical trial has reported C4.4A-ADC (BAY 1129980), a C4.4A-targeting human immunoglobulin G1 antibody (hIgG1-Ab, C4.4A-Ab), as a promising therapeutic candidate for the treatment of NSCLC and other cancers with expression of C4.4A [58], suggesting that LYPD3 may serve as a therapeutic target in tumors and LYPD3-ADC combined with ICIs may provide new strategies for NSCLC patients. Future functional studies of LYPD3 in vivo and in vitro should be carried out to elucidate its precise role in the development and immunotherapy of LUAD.

Collectively, our findings presented significant clinical implications. Firstly, we explored and discovered a novel scoring system to classify patient with different treatment strategies based on the different risk subgroups. We found that the low-risk group showed higher IPS and lower TIDE score, correlated with a better response to ICB therapy. And compared with existing biomarkers, this signature exhibited better prediction ability. These findings suggest that cellular senescence score can further stratify patient responses to immunotherapy in LUAD. Moreover, our investigation of the effects of cellular senescence on the TME could enhance understanding of the heterogeneity of responses to immunotherapy. On the other hand, uncovering how cellular senescence affects the TME can provide an opportunity for discoveries of how we can effectively remodel the immunosuppressive milieu by suppressing the process of senescence or destroying senescent cells, defined as senolytic therapies. What’s more, the GDSC database was utilized to identify multiple small-molecule drugs for LUAD. According to the estimated IC50 values, patients in low-risk subgroup showed sensitive to Roscovitine (CDKs inhibitor), Lenaidornide (TNF-α inhibitor), MK2206 (Akt 1/2/3 inhibitor) etc., helping medical staff to choose a suitable treatment method for the patient more accurately. Synergistic effects of ICIs combined with the previous drugs have been reported in cancers [59–61]. Hence, these candidate molecular drugs might possess potential efficacy for LUAD, which demonstrated promising approaches to improve immunotherapy response, especially in aged cancer patients.

Some limitations still remained in our study. Firstly, as for the CSS was constructed and validated with retrospective data, these findings need to be confirmed in a multi-center prospective study based on a larger population. Secondly, further experiments in vivo and in vitro are needed to elucidate the deeper regulatory mechanisms of cellular senescence-related genes in the development of LUAD.

## Conclusion

Our study established a novel classification for LUAD based on the mRNA expression profiles of cellular senescence-related genes. In addition, a robust cellular senescence scoring system was also developed and validated. These results might facilitate prognostic biomarker selection and provide novel insights toward personalized immunotherapy for LUAD patients in the future.

## Supplementary Information

**Additional file 1: Figure S1.** Schematic overview of the workflow employed in this study.

**Additional file 2: Figure S2.** (A) Heatmap representation of NMF clustering for cellular senescence-related genes in TCGA cohort with cluster numbers from 2–10. (B) The relationships between cophenetic, dispersion, residuals, and silhouette coefficients with respect to the number of clusters.

**Additional file 3: Figure S3.** (A) Volcano plot of the univariate Cox regression analysis showed the effect of cellular senescence-related genes on clinical prognosis in LUAD. (B) Random survival forest analysis screened 10 genes ranked by importance. (C) The Log-rank P-values of the top 20 combinations were displayed. The signature including four genes was chosen, which had the biggest -log10 P-value and relatively small number of genes. (D) The predictive value of CCS in LUAD patients of TCGA cohort (AUC: 0.690, 0.696, and 0.638 for 2, 3, and 5-years overall survival, respectively).

**Additional file 4: Figure S4.** Kaplan–Meier curves depicted the survival difference between CSS-high and CSS-low groups in different clinical subgroups including Stage I + II and Stage III + IV (A), T1 + T2 and T3 + T4 (B), N0 and N1 + N2 + N3 (C), Age ≤ 70 and Age > 70 (D), Male and Female (E), respectively.

**Additional file 5: Figure S5.** (A-D) Kaplan–Meier curves for patients with high and low expression of 4 CCS genes (C1QTNF6 (A), SQOR (B), LYPD3 (C), FAM83B (D)) in the TCGA cohort. (E) Comparisons of the expression of 4 CSS genes (C1QTNF6, SQOR, LYPD3, FAM83B) between tumor tissues and adjacent normal tissues in GEPIA2.

**Additional file 6: Figure S6.** (A) Comparisons of immune checkpoint gene expression (TIGIT, HAVCR2, PDCD1LG2, VSIR, LAG3, CD276) between CSS-low and CSS-high groups. The statistical differences were compared through the Wilcoxon test. ***P < 0.001. (B) The TMB distribution between CSS-high and CSS-low groups. (C) Kaplan–Meier curves for four patient groups stratified by CCS and TMB.

**Additional file 7: Figure S7.** The boxplots of the estimated IC50 for the top 16 significant compounds from GDSC database between high- and low-risk groups.

**Additional file 8: Table S1.** Clinical characteristics of enrolled patients in each dataset.

**Additional file 9: Table S2.** Patient characteristics in NCC cohort (n = 74).

**Additional file 10: Table S3**: A list of cellular senescence-related genes. **Table S4**: Prognostic analysis of cellular senescence-related genes using univariate Cox regression analysis. **Table S5**: A list of cellular senescence molecular pattern-related DEGs. **Table S6**: Prognostic analysis of DEGs using univariate Cox regression analysis.

## Data Availability

Gene expression profiles, clinical information and mutation data of LUAD in this study are available from the public database (TCGA, https://portal.gdc.cancer.gov/ and GEO, https://www.ncbi.nlm.nih.gov/geo).

## Abbreviations

NSCLC: Non-small-cell lung cancer
LUAD: Lung adenocarcinoma
TME: Tumor microenvironment
OS: Overall survival
TCGA: The Cancer Genome Atlas
FPKM: Fragments per kilobase million
TPM: Transcripts per million
GEO: Gene Expression Omnibus
TMA: Tissue microarray
NMF: Non-negative matrix factorization
DEGs: Differentially expressed genes
GO: Gene ontology
GSEA: Gene set enrichment analysis
GSVA: Gene set variation analysis
ssGSEA: Single sample gene set enrichment analysis
ITH: Intratumor heterogeneity
TIDE: Tumor immune dysfunction and exclusion
IPS: Immunophenoscore
GDSC: Genomics of drug sensitivity in cancer
Treg: Regulatory T cell
MDSC: Myeloid-derived suppressor cell
MHC: Major histocompatibility complex
EMT: Epithelial mesenchymal transition
CSS: Cellular senescence score
ICIs: Immune checkpoint inhibitors
