# Supplementary figures and images for: Identification and validation of cellular senescence patterns to predict clinical outcomes and immunotherapeutic responses in lung adenocarcinoma
Source: Cancer Cell Int. 2021 Dec 6;21:652. doi: 10.1186/s12935-021-02358-0 (PMC8647370; doi:10.1186/s12935-021-02358-0)

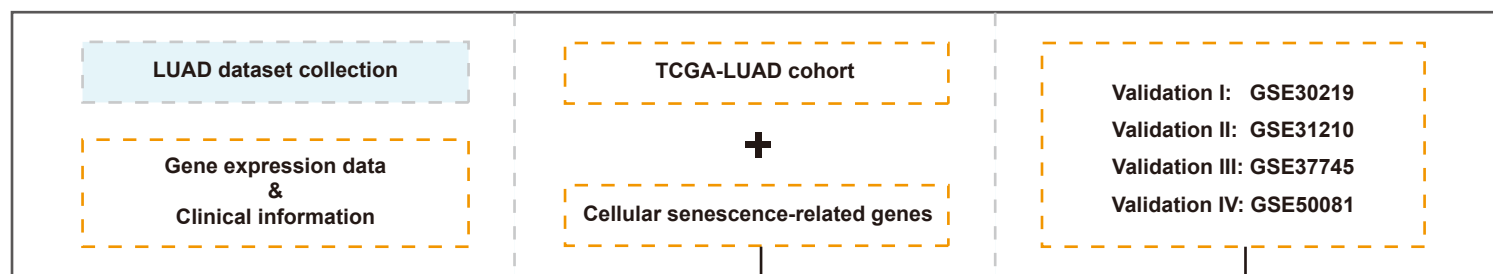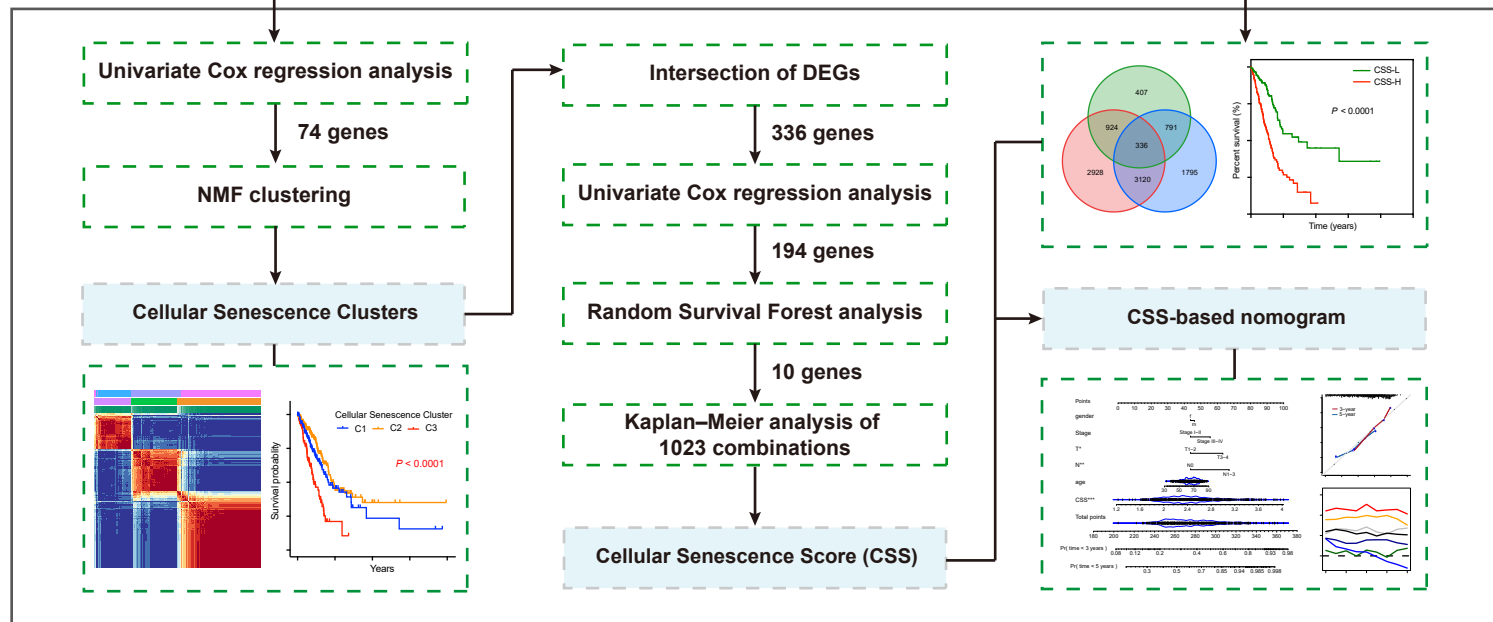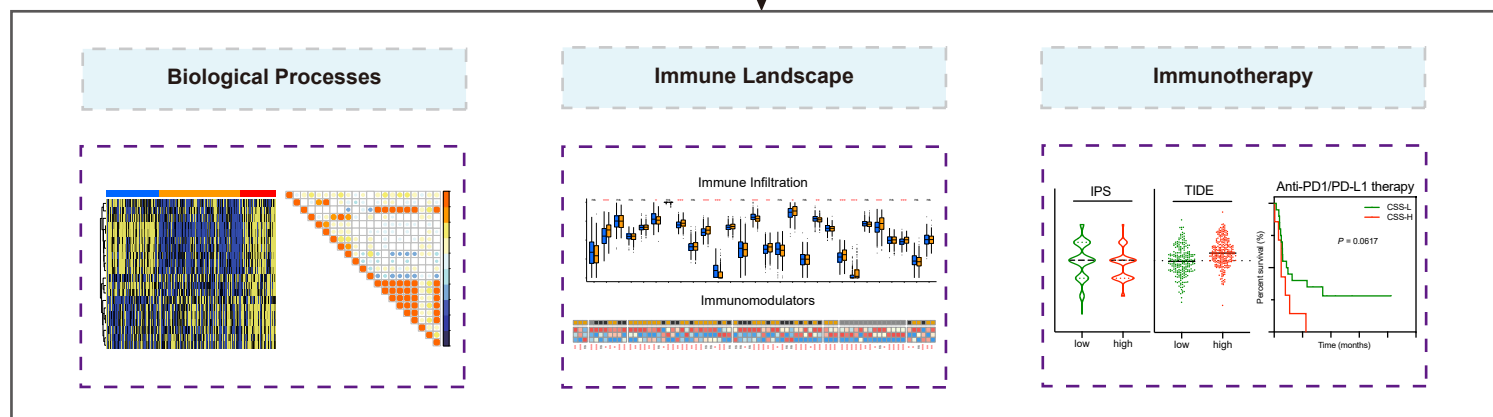

Supplement: Supplementary file 1 — Additional file 1: Figure S1. Schematic overview of the workflow employed in this study. [file 12935_2021_2358_MOESM1_ESM.pdf]

A

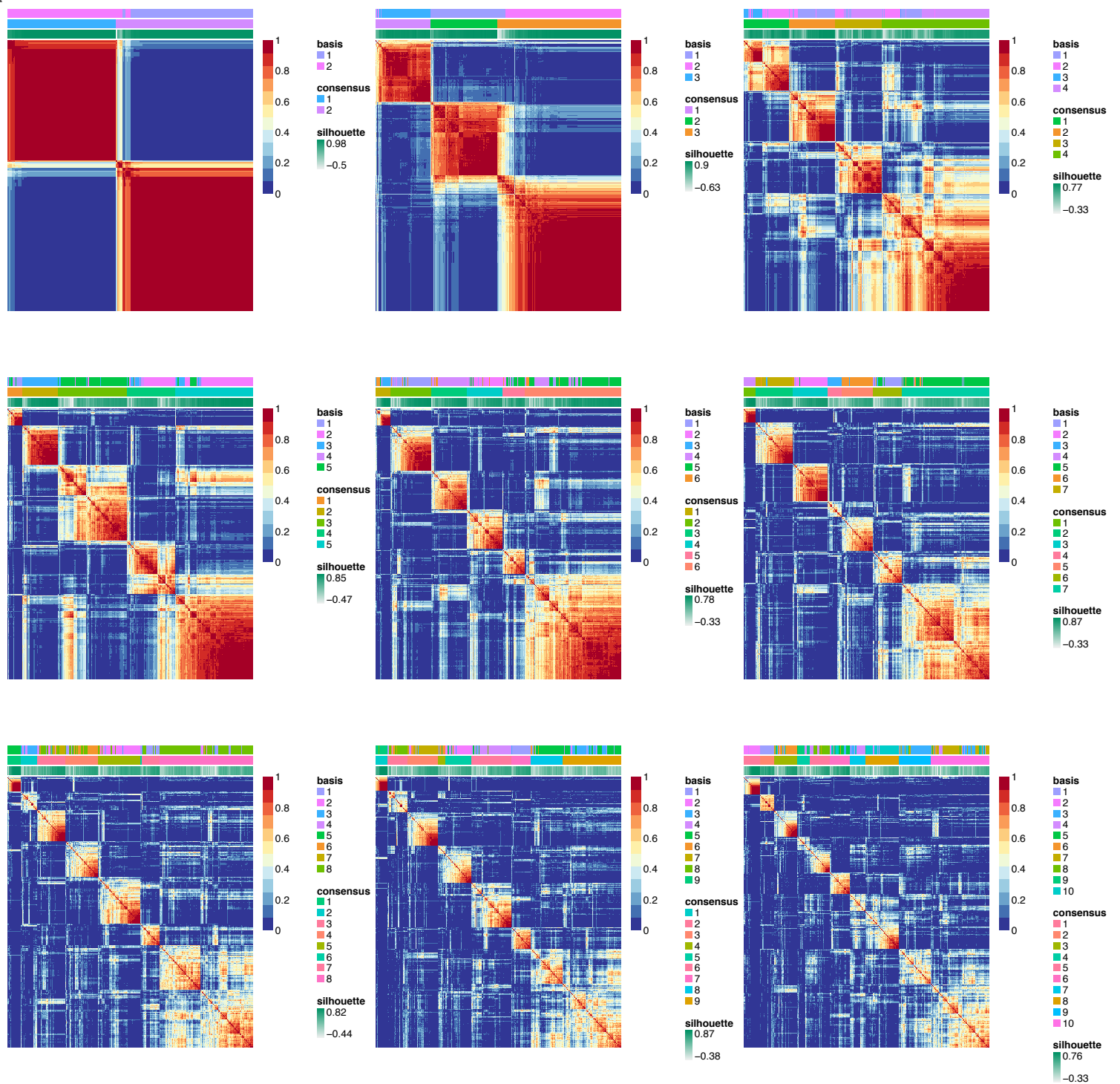

B

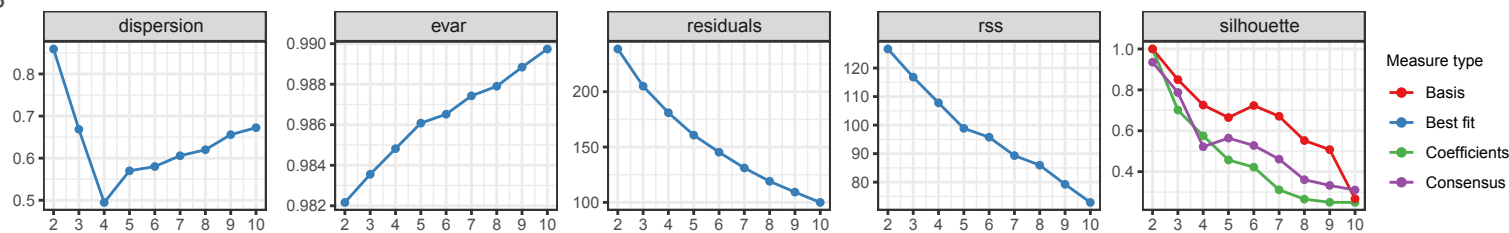

Supplement: Supplementary file 2 — Additional file 2: Figure S2. (A) Heatmap representation of NMF clustering for cellular senescence-related genes in TCGA cohort with cluster numbers from 2–10. (B) The relationships between cophenetic, dispersion, residuals, and silhouette coefficients with respect to the number of clusters. [file 12935_2021_2358_MOESM2_ESM.pdf]

A

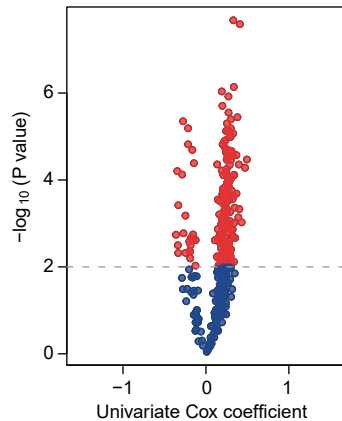

B

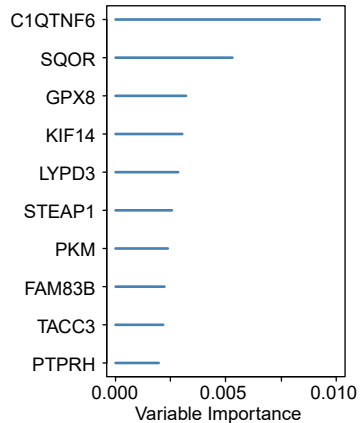

C

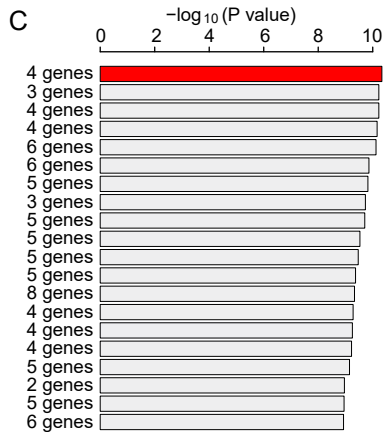

D

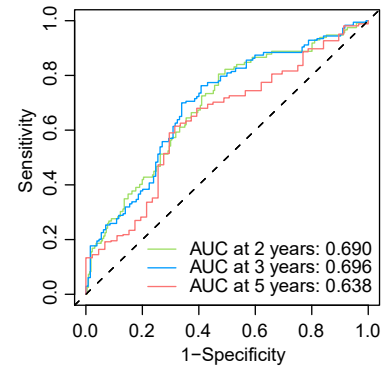

Supplement: Supplementary file 3 — Additional file 3: Figure S3. (A) Volcano plot of the univariate Cox regression analysis showed the effect of cellular senescence-related genes on clinical prognosis in LUAD. (B) Random survival forest analysis screened 10 genes ranked by importance. (C) The Log-rank P-values of the top 20 combinations were displayed. The signature including four genes was chosen, which had the biggest -log10 P-value and relatively small number of genes. (D) The predictive value of CCS in LUAD patients of TCGA cohort (AUC: 0.690, 0.696, and 0.638 for 2, 3, and 5-years overall survival, respectively). [file 12935_2021_2358_MOESM3_ESM.pdf]

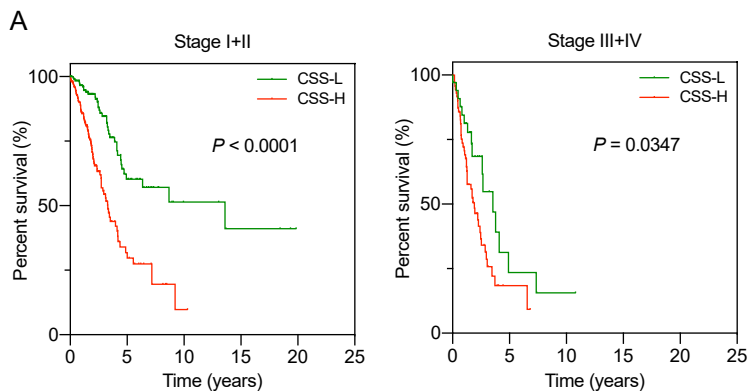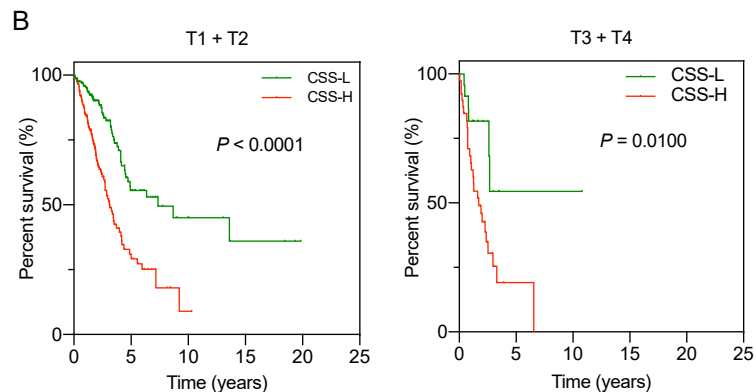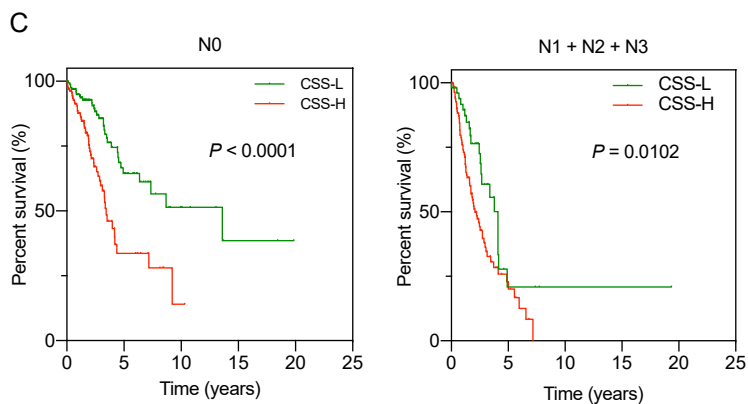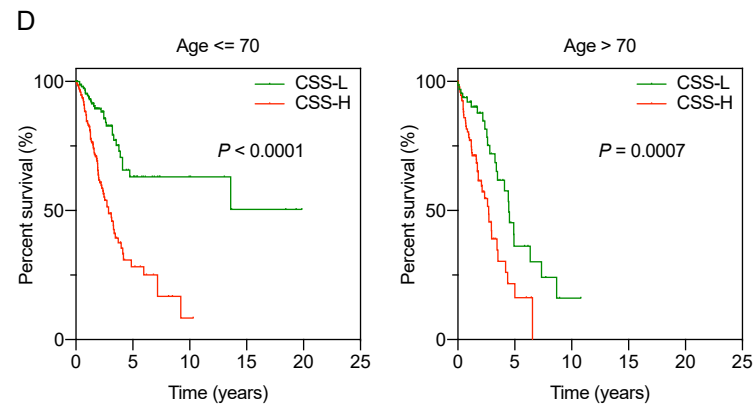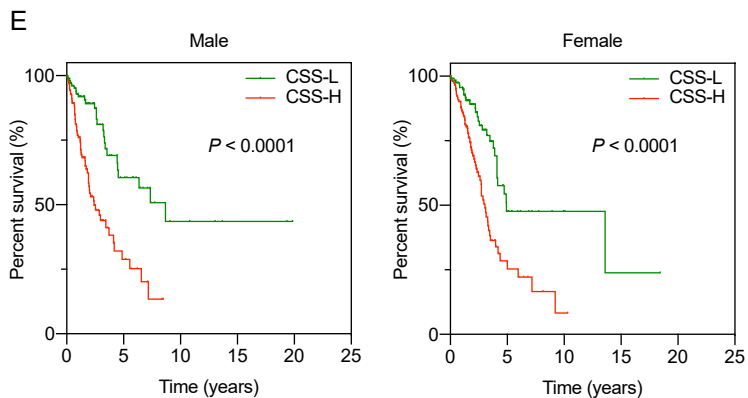

Supplement: Supplementary file 4 — Additional file 4: Figure S4. Kaplan–Meier curves depicted the survival difference between CSS-high and CSS-low groups in different clinical subgroups including Stage I + II and Stage III + IV (A), T1 + T2 and T3 + T4 (B), N0 and N1 + N2 + N3 (C), Age ≤ 70 and Age > 70 (D), Male and Female (E), respectively. [file 12935_2021_2358_MOESM4_ESM.pdf]

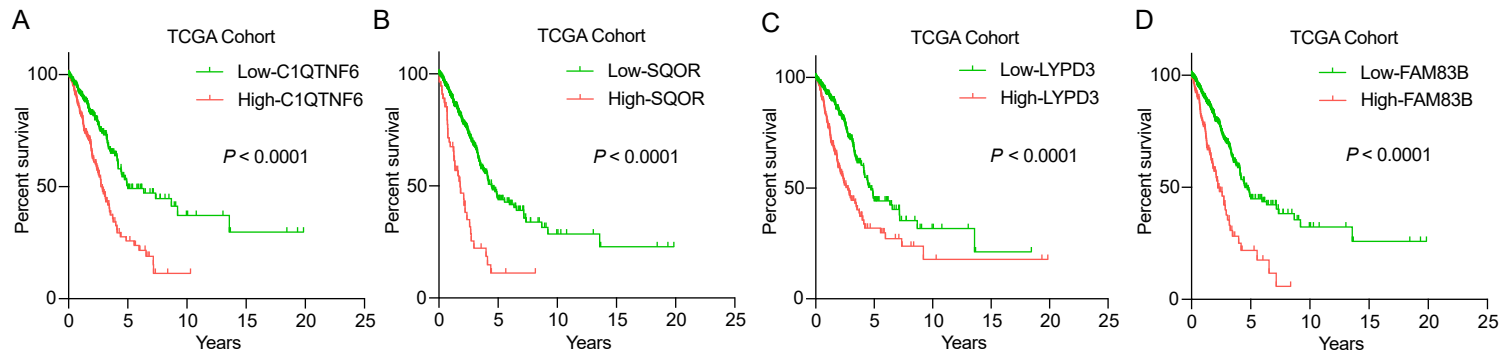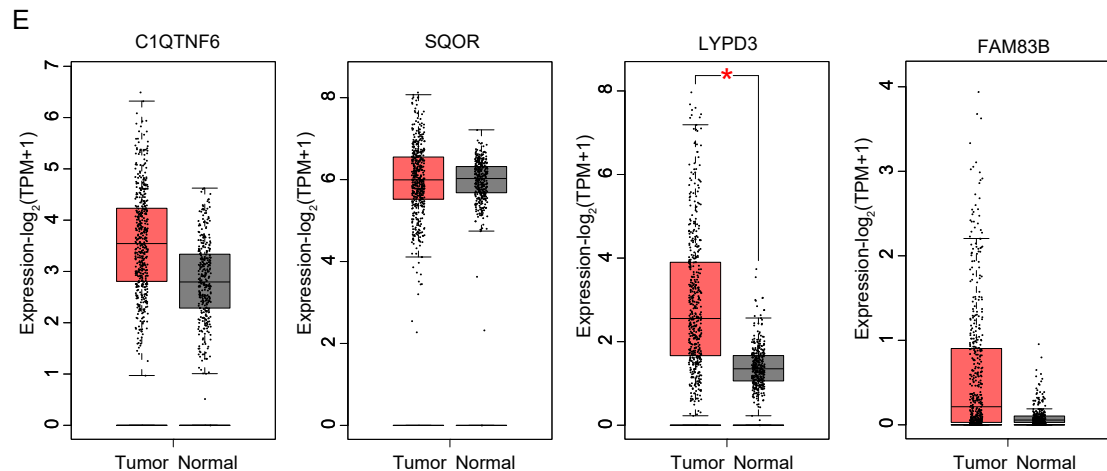

**F**

Supplement: Supplementary file 5 — Additional file 5: Figure S5. (A-D) Kaplan–Meier curves for patients with high and low expression of 4 CCS genes (C1QTNF6 (A), SQOR (B), LYPD3 (C), FAM83B (D)) in the TCGA cohort. (E) Comparisons of the expression of 4 CSS genes (C1QTNF6, SQOR, LYPD3, FAM83B) between tumor tissues and adjacent normal tissues in GEPIA2. [file 12935_2021_2358_MOESM5_ESM.pdf]

A

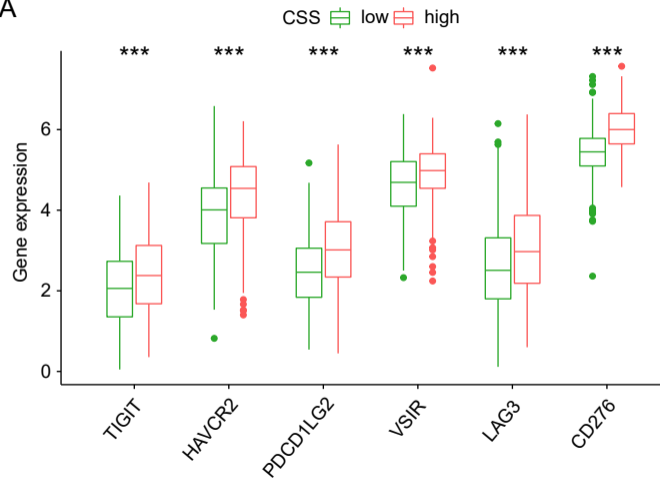

B

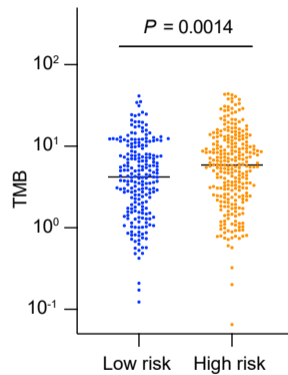

C

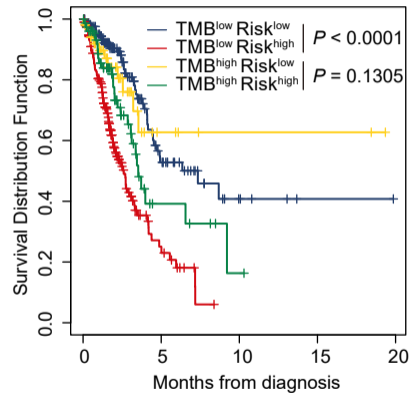

Supplement: Supplementary file 6 — Additional file 6: Figure S6. (A) Comparisons of immune checkpoint gene expression (TIGIT, HAVCR2, PDCD1LG2, VSIR, LAG3, CD276) between CSS-low and CSS-high groups. The statistical differences were compared through the Wilcoxon test. ***P < 0.001. (B) The TMB distribution between CSS-high and CSS-low groups. (C) Kaplan–Meier curves for four patient groups stratified by CCS and TMB. [file 12935_2021_2358_MOESM6_ESM.pdf]

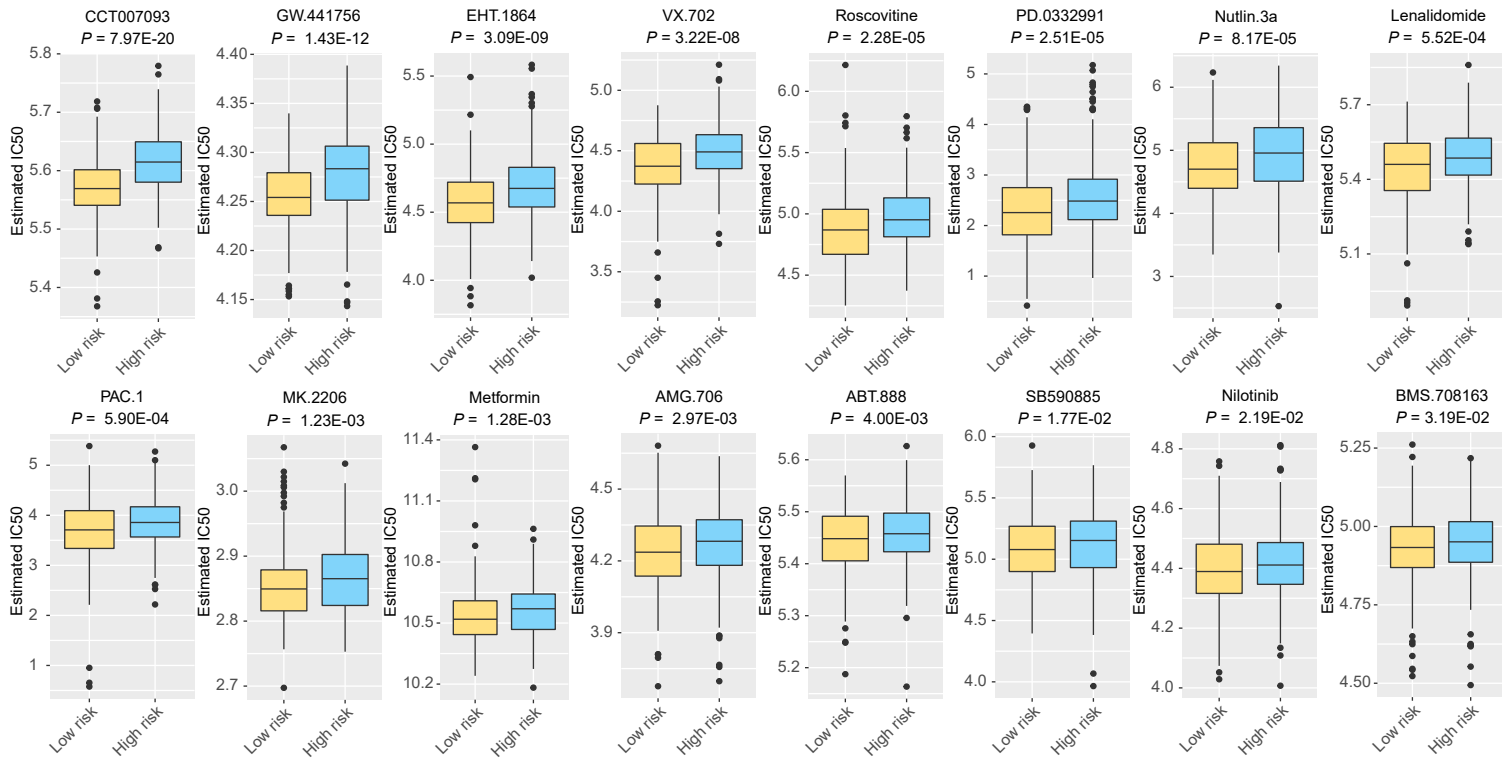

Supplement: Supplementary file 7 — Additional file 7: Figure S7. The boxplots of the estimated IC50 for the top 16 significant compounds from GDSC database between high- and low-risk groups. [file 12935_2021_2358_MOESM7_ESM.pdf]
